# Primary Hypertrophic Osteoarthropathy With Myelofibrosis

**DOI:** 10.7759/cureus.30108

**Published:** 2022-10-09

**Authors:** Muhammad Yousaf, Rubina Khan, Zaineb Akram, Qammar U Chaudhry, Raheel Iftikhar

**Affiliations:** 1 Clinical Hematology, Armed Forces Bone Marrow Transplant Center, Rawalpindi, PAK; 2 Clinical Hematology, Armed Forces Bone Marrow Transplant center, Rawalpindi, PAK; 3 Hematology, Armed Forces Bone Marrow Transplant Center, Rawalpindi, PAK; 4 Hematology and Oncology, Armed Forces Bone Marrow Transplant Center, Rawalpindi, PAK

**Keywords:** prostaglandin e2, autosomal recessive disorder, slco2a1 gene, myelofibrosis, primary hyperostotic osteoarthropathy

## Abstract

Primary hypertrophic osteoarthropathy (PHO) is a rare autosomal recessive inherited multi-system disorder characterized by a triad of pachydermia, periostosis, and clubbing. PHO was revealed to be caused by the HPGD gene producing 15-prostaglandin dehydrogenase and the SLCO2A1 gene expressing one kind of prostaglandin transporter. It is primarily a benign disorder, but coexisting myelofibrosis can lead to clinically significant cytopenias. In this case report, we present the case of a 21-year-old boy with a history of transfusion-dependent anemia and a progressive increase in transfusion requirements over the course of seven years. On basis of the patient’s medical history, family history, and clinical examination genetic testing was done. The patient was found to have homozygous c.664G>A (p. Gly222Arg) mutation in the SLCO2A1 gene; confirming the diagnosis of PHO.

## Introduction

Primary hypertrophic osteoarthropathy (PHO), also known as Touraine Solente Gole (TSG) syndrome or Pachy-dermoperiostosis (PDP), is an inherited, autosomal recessive, rare, multi-system disorder characterized by a triad of thickening of the skin (pachydermia), abnormal growth of bone and connective tissue (periostosis) and digital clubbing (acropachia) [1]. In 2008, the mutation in the HPGD gene, which codes for the 15-hydroxyprostaglandin dehydrogenase (15-PGDH) was determined to be the major cause of PHO. In 2012 another pathogenic gene was identified to be the solute carrier organic anion transporter family member 2A1 (SLCO2A1), which encodes the prostaglandin transporter [2]. The manifestation usually begins at puberty and progresses gradually over years, with a wide spectrum of diverse radiological and clinical features. Several cases of PHO have been reported to be complicated by life-threatening anemia and gastrointestinal complications (such as hypertrophic gastropathy) [3]. Anemia in patients can be caused by various mechanisms, including the development of serum erythropoiesis inhibitors, bone marrow failure, and blood loss mainly from the gastrointestinal tract [4].

## Case presentation

**Figure 1:**
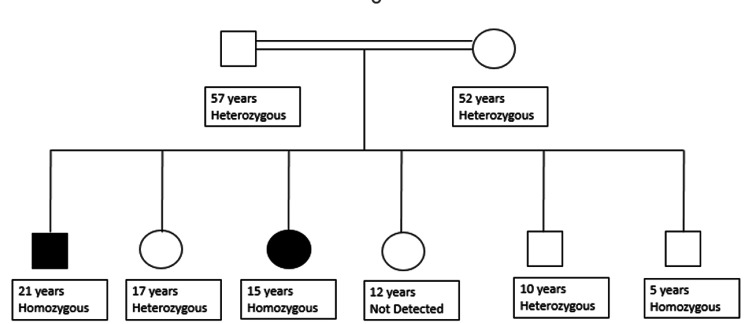
Family Tree

**Figure 2:**
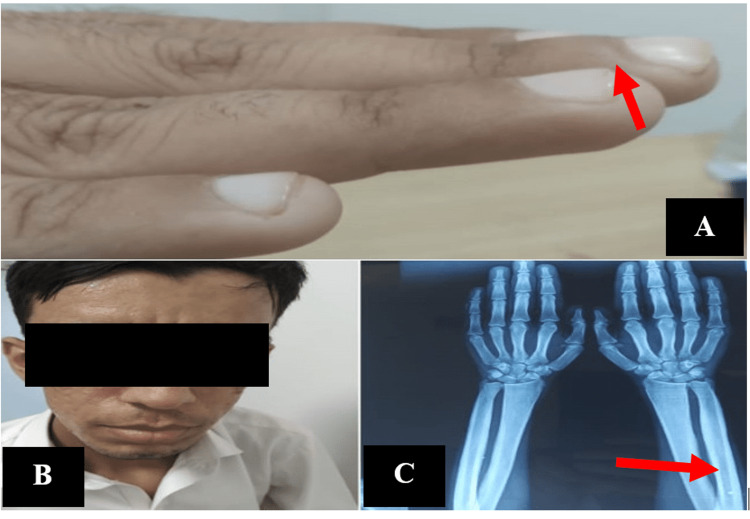
A: Patient showing clubbing, B: Patient showing frontal bossing, protruding jaw, C: Patient showing increased bone density

A 21-year-old male, a resident of Punjab, Pakistan, a tailor by profession, presented to a local hospital with a seven-year history of intermittent low-grade fever (not associated with rigors and chills), lethargy, generalized weakness, excessive sweating, body aches and joint pains affecting large joints i.e., bilateral wrist and knee joints. The patient had five siblings and the parents were in a consanguineous marriage (Figure 1). A general physical examination revealed conjunctival pallor, coarse wrinkles on the forehead, prominent nasolabial folds, acne on the face, jaw protrusion, swollen bilateral wrist joints, and grade I hand clubbing (Figure 2). His cardiovascular and respiratory examinations were unremarkable, and the abdominal examination indicated hepatosplenomegaly, with the liver palpable 2 cm below the costal margin (BCM) and being firm, non-tender with smooth edge, while the spleen was palpable 4 cm BCM (non-tender, soft). His medical history was significant for transfusion-dependent anemia requiring ~90 units of red cell concentrates (RCC) during the preceding six years. Since 2020, his monthly RCC transfusion demand had climbed from two to three.

His five-year-old sister likewise had a history of similar symptoms and transfusion dependency. Clinical examination revealed conjunctival pallor, frontal bossing, protruding jaw, grade II clubbing in hands, and dark discoloration of the skin with a few dry lesions on the anterior portion of bilateral lower limbs. A dermatologist was taken on board for the lesions on lower limbs, which were labeled as pachydermoperiostosis. The systemic examination proved uneventful. She has had t 11-12 RCCs in the past five years (~1 RCC/ six months). 

His blood complete picture (CP) showed, Hemoglobin (Hb): 4.7 g/dl, mean corpuscular volume (MCV): 96 fl, white blood cell (WBC): 3.1 x103/L, absolute neutrophil count (ANC): 2.6 x103/L, platelets: 47 x109/L on Sysmex Hematology Analyzer (also confirmed manually). The peripheral blood smear revealed anisocytosis, microcytosis, hypochromia, tear drop cells, two nucleated red blood cells (NRBC) per 100 WBCs, and a reticulocyte count of 1.5%. A diluted bone marrow sample was received with no evaluable fragment, whereas a trephine biopsy showed a hypercellular marrow with overall cellularity of ~80% and megakaryocytes forming clusters. A grade 2 marrow fibrosis (as per WHO marrow fibrosis grading), suggestive of overt myelofibrosis was also observed. Myelofibrosis was further examined for its underlying etiology. Polymerase chain reaction (PCR) for JAK2 (V617F), CALR (exon 9), cMPL (exon 10), and BCR-ABL1 were carried out, and no mutation was detected. The autoimmune profile [including antinuclear antibodies (ANA), extractable nuclear antigen antibodies panel, anti-cyclic citrullinated peptide (CCP), rheumatid Arthritis (RA) factor] was negative. Bone scan showed diffusely increased tracer uptake in the appendicular skeleton. All these findings were consistent with myelofibrosis. 

On the basis of the patient’s history, family history, and clinical examination suspicion of hypertrophic osteoarthropathy (HOA) was raised and genetic testing was advised. The patient was found to be homozygous for the c.664G>A (p. Gly222Arg) mutation in SLCO2A1 gene. Additional variants of uncertain significance were found in genes COL10A1, IFT172, MESDC2, PCNT, RUNX2, SQSTM1, TONSL, TRIM37, WDR35, and WDR60. The family was also screened for the detected mutation and the results are shown in Table 1.

**Table 1:** Genetic Screening results of the family

Family	Gene	Zygosity	Symptoms
Father 57 years, Male	SLCO2A1(p.Gly222Arg)	Heterozygous	Asymptomatic
Mother 52 years, Female	SLCO2A1(p.Gly222Arg)	Heterozygous	Asymptomatic
Patient 21 years, Male	SLCO2A1(p.Gly222Arg)	Homozygous	Transfusion Dependency
Sibling 17 years, Female	SLCO2A1(p.Gly222Arg)	Heterozygous	Asymptomatic
Sibling 15 years, Female	SLCO2A1(p.Gly222Arg)	Homozygous	Transfusion Dependency
Sibling 12 years, Female	SLCO2A1(p.Gly222Arg)	Not detected	Asymptomatic
Sibling 10 years, Male	SLCO2A1(p.Gly222Arg)	Heterozygous	Asymptomatic
Sibling 5 years, Male	SLCO2A1(p.Gly222Arg)	Homozygous	Asymptomatic

Based on all the above features, a diagnosis of primary idiopathic PHO with an unusually severe complication of myelofibrosis was determined. He was started on erythropoietin and cyclooxygenase-2 (COX 2) inhibitors, causing a reduction of transfusion dependency.

## Discussion

PHO is a rare genetic disorder majorly characterized by abnormal bone formation and dermatosis and is poorly understood [5]. The pathogenic association of PHO and myelofibrosis remains poorly known. Numerous mediators, including platelet-derived growth factors (PDGF), TGF-beta 1, and vascular endothelial growth factor (VEGF), secreted by platelets and megakaryocyte alpha-granules have been identified as stimuli for increased fibroblastic activity [6]. The key feature in the pathogenesis of PHO is the failure of prostaglandin E 2 (PGE2) degradation. Several genes, such as HPGD, prostaglandin-endoperoxidase synthase (PTGS) 1, PTGS2, prostaglandin E synthase (PTGES), PTGER1, prostaglandin E receptor (PTGER) 2, PTGER3, PTGER4, SLCO2A1, SLCO3A1, SLCO4A1, and PTGR2, are involved in the biosynthesis and signaling pathway of PGE2 [7]. A recent analysis suggested that patients with prostaglandin transporter SLCO2A1 mutations are more likely to develop myelofibrosis [8].

According to the best of our knowledge, only 22 PHO cases with myelofibrosis have been reported to date, nine (40.9%) were reported from Asian countries [9]. Our patient had a long history of fatigue and other anemia-associated symptoms, and developed transfusion dependency for the last year, suggesting that hematopoiesis was well compensated at the beginning of the disease. No standard treatment for PHO patients with myelofibrosis has been established. Severe anemia can be treated with blood transfusions. Few cases report successful treatment of anemia with steroids and hematinic [10].

## Conclusions

Although PHO is primarily a benign disorder, but the coexisting myelofibrosis can lead to clinically significant cytopenia. To avoid missing the diagnosis, we recommend taking detailed history notes from such patients along with regular clinical and laboratory examinations on a regular basis.
